# Seroprevalence of hepatitis B, hepatitis C, human immunodeficiency virus, *Treponema pallidum*, and co-infections among blood donors in Kyrgyzstan: a retrospective analysis (2013–2015)

**DOI:** 10.1186/s40249-017-0255-9

**Published:** 2017-02-21

**Authors:** Bakyt B. Karabaev, Nurgul J. Beisheeva, Aiganysh B. Satybaldieva, Aikul D. Ismailova, Frank Pessler, Manas K. Akmatov

**Affiliations:** 1 Republican Blood Centre, Bishkek, Kyrgyzstan, Chingiz Aitmatov Ave 60, 720044 Bishkek, Kyrgyzstan; 2 Republican AIDS Centre, Bishkek, Kyrgyzstan, Logvinenko Str. 8, 720040 Bishkek, Kyrgyzstan; 3 TWINCORE, Centre for Experimental and Clinical Infection Research, Feodor-Lynen-Str. 7, 30625 Hannover, Germany; 4 Helmholtz Centre for Infection Research, Inhoffenstr. 7, 38124 Braunschweig, Germany; 5 Centre for Individualized Infection Medicine, c/o CRC Hannover, Feodor-Lynen-Str. 15, 30625 Hannover, Germany

**Keywords:** Hepatitis B, Hepatitis C, Human immunodeficiency virus, *T. pallidum*, Co-infections, Prevalence, Blood donors, Kyrgyzstan

## Abstract

**Background:**

Post-Soviet Kyrgyzstan has experienced a major surge in blood-borne infections, but data from adequately powered, up-to-date studies are lacking. We thus examined a) the seroprevalences of hepatitis B virus surface antigen (HBsAg), HIV-1 p24 antigen and antibodies against hepatitis C virus (anti-HCV), human immunodeficiency viruses (anti-HIV-1/2, HIV-1 group O), and *Treponema pallidum* among blood donors in Kyrgyzstan and assess their distribution according to sex, age, and provinces of residence; b) trends in the respective seroprevalences; and c) co-infection rates among the pathogens studied.

**Methods:**

Serological screening was performed on 37 165 blood donors at the Republican Blood Centre in Bishkek, Kyrgyzstan, between January 2013 and December 2015. We applied poststratification weights to control for sampling bias and used logistic regression analyses to examine the association of seropositivity and co-infections with sex, age, provinces of residence, and year of blood donation.

**Results:**

Twenty nine thousand and one hundred forty-five (78%) donors were males and 8 020 (22%) were females. The median age was 27 years (range: 18 – 64). The prevalences of HBsAg, anti-HCV, HIV (p24 Ag and anti-HIV), and anti-*T. pallidum* were 3.6% (95%*CI*: 3.4 – 3.8%), 3.1% (3.0 – 3.3%), 0.78% (0.69 – 0.87%), and 3.3% (3.1 – 3.5%), respectively. Males were more likely to be seropositive for HBsAg than females (*OR*: 1.63; 95%*CI*: 1.40 – 1.90), but less likely to be seropositive for anti-HCV (0.85; 0.74 – 0.98) and HIV (0.65; 0.49 – 0.85). Prevalences were lower in the capital than in the other provinces. There was a decreasing trend in the seroprevalences of HBsAg, anti-HCV, and anti-*T. pallidum* from 2012 to 2015 (*P*-value for trend, *P* = 0.01, *P* < 0.0001, *P* < 0.0001, respectively), while the seroprevalence of HIV increased (*P* = 0.049). One hundred eighty donors (0.48%) were seropositive for multiple infections. The highest co-infection rate was observed between anti-*T. pallidum* and HBsAg (6.0%), followed by anti-HCV and anti-*T. pallidum* (5.2%), and HIV and anti-HCV (4.9%).

**Conclusions:**

The data suggest that Kyrgyzstan can be reclassified from high to lower-intermediate HBsAg endemicity, whereas the high HIV prevalence with a rising trend is an alarming finding that needs to be urgently addressed by public health authorities. The observed co-infections suggest common risk factors but also common preventive interventions.

**Electronic supplementary material:**

The online version of this article (doi:10.1186/s40249-017-0255-9) contains supplementary material, which is available to authorized users.

## Multilingual abstracts

Please see Additional file 1 for translation of the abstract into the five official working languages of the United Nations.

## Background

Hepatitis B virus (HBV), hepatitis C virus (HCV), human immunodeficiency virus (HIV), and *Treponema pallidum* still cause high burdens of disease in many countries, especially in developing countries. For example, 184 million and 248 million individuals worldwide are chronic carriers of HCV and HBV, respectively [1, 2]. In addition, around six million individuals are infected with *T. pallidum* [3] and 37 million individuals are living with HIV/acquired immunodeficiency syndrome (AIDS) globally [4].

The transmission of these infectious agents comprises various routes, including transmission from mother to infant (vertical transmission), sexual transmission, exposure to infected blood due to using contaminated needles and syringes, and the transfusion of infected blood or its components. The latter route is very important since a blood transfusion is a frequent therapeutic procedure, with around 108 million units of donated blood collected every year worldwide [5]. Thus, the World Health Organization (WHO) recommends that all blood donations should be screened for selected infections prior to use and that screening should be mandatory for HBV, HCV, HIV, and *T. pallidum* [5]. Evaluation of data on the prevalence of these infections among blood donors may provide information about the epidemiology of these infections in the general population [1, 6].

Kyrgyzstan is one of the 15 former Soviet republics that became independent in 1991 after the collapse of the Soviet Union. Since then, the country has experienced a deep political, economic, and societal crisis, which has resulted in deteriorating health among the population, including increased morbidity and mortality due to infectious diseases [7, 8]. Kyrgyzstan is classified as a lower middle-income country with around one third of the population living below the poverty line.

Up until now, only a few studies have assessed the prevalences of infectious diseases in Kyrgyzstan. Most of them have been conducted in selected subpopulations or featured small sample sizes; for infections such as syphilis there are no up-to-date data at all. The recently published data on global prevalence of chronic HBV classified Kyrgyzstan as a country with high endemicity [1]. However, this classification was based on a single publication from 1992 [9]. The prevalences of HIV in the general Kyrgyz population seem to be low; however, we are not aware of any study that has assessed the prevalence of HIV in the general population or among blood donors.

It is estimated that the prevalence of HIV in adults is around 0.1% in Kyrgyzstan [10]. Kyrgyzstan is one of the countries with the fastest growing HIV epidemics along with Ukraine, Russia, and Uzbekistan. Regarding HCV, Kyrgyzstan was classified as a country with a high prevalence (>3.5%) in 2005 [2]. After the breakdown of the Soviet Union, the incidence of syphilis increased dramatically in most former Soviet republics including Kyrgyzstan [11]. It began to decline in 1997 but current data are lacking.

In addition, little is known about co-infection rates of the abovementioned infections in Kyrgyzstan. This information is important to have, as co-infections may lead to more severe disease outcomes and faster disease progression. For example, HIV infection may accelerate the course of HBV and HCV infection by leading to faster development of fibrosis and cirrhosis [12, 13]. Treatment of co-infections can also be problematic as it is associated with an increased risk of side effects due to drug-drug interactions and poor compliance if treatment is aborted early, as in the case of HIV/HCV treatment [14]. Lastly, recognition of co-infections is important because shared transmission routes and mechanisms may suggest common preventive interventions.

With all this in mind, the aims of the present study were to a) examine the seroprevalences of hepatitis B surface antigen (HBsAg), antibodies against HCV and *T. pallidum*, and HIV-1 p24 antigen plus anti-HIV antibodies among blood donors in Kyrgyzstan according to sex, age, and provinces of residence; b) examine trends in the respective seroprevalences over time; and c) assess co-infection rates among the pathogens studied.

## Methods

### Sampling

We used data of 37 165 adult volunteers who donated blood at the Republican Blood Centre in Bishkek, Kyrgyzstan between January 2013 and December 2015. Kyrgyzstan is a former Soviet republic located in Central Asia. The population was approximately 5,940,000 in 2015 [15].

Physicians interviewed the potential donors before the blood donation. A physician collected information on the presence of acute and chronic infectious diseases, chronic noncommunicable diseases, and risk factors for blood-transmissible diseases (e.g., men who have sex with men [MSM] or sex workers) during face-to-face interviews. In addition, height, weight, blood pressure, and body temperature were measured. Only individuals without a history of the abovementioned diseases or risk factors for blood-transmissible diseases were allowed to donate blood. There were no financial or other incentives for blood donation. In the case of serial blood donations during the study period, we only included one randomly selected serological result.

### Laboratory analysis

Enzyme-linked immunoassay (ELISA) tests were performed using a microplate spectrophotometer (Thermo Scientific Multiskan FC, Vantaa, Finland). The kits DS-EIA-HBsAg, EIA anti-HCV, DS-EIA-HIV-AgAb-SCREEN, and EIA-anti-LUES-GM manufactured by RPC Diagnostic systems (Nizhny Novgorod, Russia) were used to test for the presence of HBsAg, and HIV-1 p24 antigen; and for antibodies against HCV (anti-HCV, for HCV core antigen, NS3Ag, NS4Ag, and NS5-Ag) and HIV (anti-HIV-1/2, HIV-1 group O); and *T. pallidum* (anti-*T. pallidum*), respectively*.* Seropositivity for HIV was defined as detection of HIV-1 p24 antigen and/or anti-HIV antibodies, as the read-out from the HIV test can come from reactivity with either p24 Ag or anti-HIV antibodies. All positive samples were retested once using the same ELISA assays and the same blood sample.

Samples were considered positive if results were positive in both tests. Overall, the ELISA tests used have high sensitivity and specificity (see Table 1) and have been licensed for use by the Kyrgyzstan Ministry of Health.

**Table 1:** ELISA kits used in the study

Kit names	Infection	Sensitivity	Specificity	Detection limits	Manufacturer’s catalogue number	Reference
DS-EIA-HBsAg	HBV	100%	99%	0.05 IU/ml	B-1154	[37]
EIA anti-HCV	HCV	100%	100%	NQ^a^	C-153	[38]
DS-EIA-HIV-AgAb-SCREEN	HIV	100%	99.6%	0.05 IU/ml	I-1654	[39]
EIA-anti-LUES-GM	Syphilis	99.4%	99.6%	0.0047 IU/ml	L-155	[40]

^a^Numerically not quantified, but significantly below the optical density corresponding to the definition of a positive test

### Statistical analysis

Since the proportion of male and younger donors was higher in the sample than in the general population, we applied poststratification weights using sex and 10-year age groups. The sex and age distribution of the Kyrgyz population was taken from the last census conducted in 2009 [16]. We then calculated weighted sex- and age-specific prevalence rates of HBV, HCV, HIV, and *T. pallidum*. Furthermore, we used linear-by-linear association from the chi-square test to examine trends in the seroprevalence rates. As a next step, we applied logistic regression analysis to examine odds of seropositivity by adjusting for donors’ sex, age, province of residence, and year of blood donation.

Four separate models (HBV, HCV, HIV, and *T. pallidum*) were created. A separate logistic regression model was created to examine the odds of having co-infections, adjusting for sex, age, province of residence, and year of blood donation. Finally, we examined the effects of co-infections on the respective seropositivities using a logistic regression model with, for example, HBV as a dependent and *T. pallidum* as an independent variable or vice versa, and adjusted for sex and age.

Analyses were performed with IBM SPSS Statistics for Windows, version 19 (IBM Corporation, Armonk, NY, USA). The Venn diagram (see Fig. 2) was drawn using the package ‘VennDiagram’ in the R Foundation for Statistical Computing, version 3.0.2.

## Results

Of the 37 165 blood donors, 29 145 (78%) were males and 8 020 (22%) were females. The median age was 27 years (range: 18 – 64). More than half (54%) were from the age group ‘20–29 years’. Almost half of the donors (49%) lived in the capital, Bishkek (see Table 2).

**Table 2:** Descriptive characteristics of the study population

Characteristics	Unweighted (*n*)	Percent (%)
Sex		
Male	29 145	78.4
Female	8 020	21.6
Median age in years (range, IQR)		27 (18 – 64; 22 – 36)
Age group		
< 20 years	1 679	4.0
20 – 29 years	20 213	54.4
30 – 39 years	8 547	23.0
40 – 49 years	4 457	12.0
50 – 59 years	2 278	6.1
> 60 years	191	0.5
Province of residence		
Bishkek (capital)	18 086	48.7
Chuy province	10 786	29.0
Issyk-Kul province	2 574	6.9
Talas province	961	2.6
Naryn province	2 050	5.5
Jalal-Abad province	1 305	3.5
Osh province	784	2.1
Batken province	614	1.7
Foreign country	4	0.01
Year of blood donation		
2013	12 086	32.5
2014	12 437	33.5
2015	12 642	34.0
Total	37 165	100

*IQR* interquartile range

**Fig. 1:**
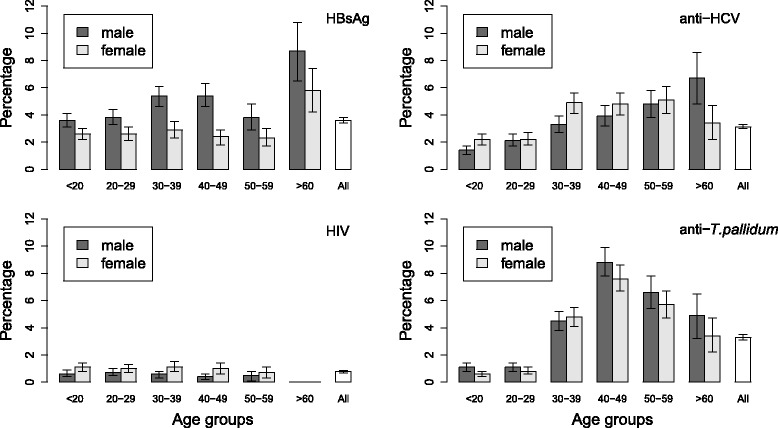
Prevalences of HBV, HCV, HIV, and *T. pallidum*, by sex and age groups

The prevalences of HBsAg, anti-HCV, HIV, and anti-*T. pallidum* were 3.6% (95%*CI*: 3.4 – 3.8%), 3.1% (3.0 – 3.3%), 0.78% (0.69 – 0.87%), and 3.3% (3.1 – 3.5%), respectively. Males were more likely to be seropositive for HBsAg in all age groups, while females were more likely to be seropositive for anti-HCV and HIV (see Fig. 1, and Table 3). The odds of seropositivity for HBsAg and anti-HCV increased with age (see Fig. 1). The highest odds of seropositivity for *T. pallidum* were observed in the age group ‘40-49 years’ and decreased in later years. Regional differences in seroprevalences were also observed: for most infections, they were lower in the capital than in the other provinces (see Table 3). There was a decreasing trend with time in the seroprevalences of HBsAg (*P* for trend, *P* = 0.01), anti-HCV (*P* < 0.0001) and anti-*T. pallidum* (*P* < 0.0001). The seroprevalence of HIV was higher in 2014 and 2015 than in 2013 (*P* for trend, *P* = 0.049).

**Table 3:** Associations of seropositivity and co-infections with sex, age, province of residence, and year of blood donation (results of five multivariable logistic regression models)

	AORs and 95%*CI*s^a^				
Variables	HBV	HCV	HIV	*T. pallidum*	Co-infections^b^
Sex					
Male	1.63 (1.40 – 1.90)	0.85 (0.74 – 0.98)	0.65 (0.49 – 0.85)	1.09 (0.94 – 1.26)	1.64 (1.09 – 2.48)
Female	ref.	ref.	ref.	ref.	ref.
Age group					
< 20 years	1.02 (0.76 – 1.39)	0.84 (0.55 – 1.27)	0.83 (0.46 – 1.51)	0.80 (0.45 – 1.41)	0.84 (0.20 – 3.49)
20 – 29 years	ref.	ref.	ref.	ref.	ref.
30 – 39 years	1.40 (1.24 – 1.58)	1.70 (1.46 – 1.97)	0.88 (0.65 – 1.20)	4.44 (3.75 – 5.26)	4.20 (2.72 – 6.48)
40 – 49 years	1.37 (1.17 – 1.60)	1.93 (1.62 – 2.30)	0.75 (0.49 – 1.15)	8.95 (7.54 – 10.62)	9.32 (6.07 – 14.29)
50 – 59 years	1.00 (0.78 – 1.28)	2.25 (1.82 – 2.79)	0.72 (0.41 – 1.28)	6.54 (5.26 – 8.14)	7.10 (4.13 – 12.21)
> 60 years	2.35 (1.35 – 4.09)	2.24 (1.17 – 4.27)	NA	4.43 (2.15 – 9.13)	7.91 (1.87 – 33.43)
Province of residence					
Bishkek (capital)	ref.	ref.	ref.	ref.	ref.
Chuy province	1.00 (0.88 – 1.13)	0.96 (0.84 – 1.11)	0.73 (0.53 – 1.01)	1.07 (0.93 – 1.23)	0.92 (0.65 – 1.29)
Issyk-Kul province	0.86 (0.68 – 1.09)	0.73 (0.54 – 0.97)	1.65 (1.10 – 2.46)	1.57 (1.24 – 1.98)	0.61 (0.28 – 1.33)
Talas province	1.41 (1.05 – 1.89)	0.75 (0.47 – 1.17)	0.72 (0.29 – 1.78)	0.80 (0.50 – 1.26)	NA
Naryn province	1.09 (0.86 – 1.38)	0.85 (0.77 – 1.15)	1.53 (0.97 – 2.41)	1.44 (1.12 – 1.87)	1.24 (0.67 – 2.30)
Jalal-Abad province	1.80 (1.42 – 2.28)	1.08 (0.87 – 1.92)	0.96 (0.48 – 1.89)	2.03 (1.54 – 2.68)	1.66 (0.85 – 3.24)
Osh province	1.70 (1.26 – 2.30)	1.29 (1.07 – 2.32)	0.69 (0.26 – 1.89)	1.54 (1.04 – 2.27)	2.19 (1.05 – 4.59)
Batken province	2.31 (1.71 – 3.13)	0.71 (0.40 – 1.26)	1.42 (0.62 – 3.24)	1.38 (0.89 – 2.16)	0.96 (0.30 – 3.08)
Year of blood donation					
2013	ref.	ref.	ref.	ref.	ref.
2014	0.95 (0.84 – 1.08)	0.72 (0.62 – 0.84)	5.22 (3.48 – 7.83)	0.86 (0.74 – 1.00)	0.96 (0.66 – 1.41)
2015	0.79 (0.69 – 0.90)	0.78 (0.67 – 0.90)	2.71 (1.74 – 4.20)	1.00 (0.86 – 1.16)	1.25 (0.871.79)

*NA* not assessed

^a^Adjusted for all variables in the table

**Fig. 2:**
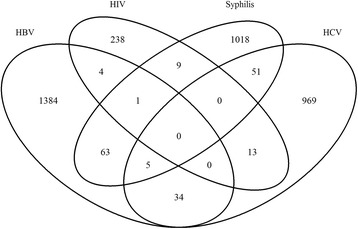
Venn diagram showing overlap of HBV, HCV, HIV, and *T. pallidum* seropositivity

^b^In total, 180 (0.48%) blood donors were positive for multiple infections (see Fig. 2)

The co-infection rates for HBV, HCV, HIV, and *T. pallidum* are presented in Table 4. The highest co-infection rate was observed between *T. pallidum* and HBV (6.0%), followed by HCV and *T. pallidum* (5.2%), and HIV and HCV (4.9%).

**Table 4:** Co-infection rates of HBV, HCV, HIV, and *T. pallidum* ^a^

Co-infection rates	Total positive (*n*)	% positive
HBV with HCV	39	2.6
HCV with HBV	39	3.6
HBV with HIV	5	0.34
HIV with HBV	5	1.9
HBV with *T. pallidum*	69	4.6
*T. pallidum* with HBV	69	6.0
HCV with HIV	13	1.2
HIV with HCV	13	4.9
HCV with *T. pallidum*	56	5.2
*T. pallidum* with HCV	56	4.9
HIV with *T. pallidum*	10	3.8
*T. pallidum* with HIV	10	0.87

^a^ Co-infection rates were calculated twice for each infection combination with the corresponding denominators; i.e., of all HBV-infected individuals, 2.6% had HCV co-infection, but of all HCV-individuals, 3.6% were co-infected with HBV

The risks of HBV and HCV seropositivity were significantly higher among blood donors who were also seropositive for *T. pallidum*. The risk of acquiring HCV seropositivity was 1.83 times higher for donors positive for HIV infection (see Table 5). In total, 180 (0.48%) blood donors were found to be seropositive for more than one infection (see Fig. 2). One person (male, age group 30 – 39 years) was positive for three infections: HBV, HIV, and *T. pallidum*. In five individuals (all males between 39 and 56 years of age), results were positive for HBV, HCV, and *T. pallidum*.

**Table 5:** Effects of co-infection on the respective seropositivities*

	HCV	HIV	*T. pallidum*
HBV	0.89 (0.64 – 1.23) *P* = 0.48	0.48 (0.20 – 1.17) *P* = 0.11	1.46 (1.14 – 1.88) *P* = 0.003
HCV	-	1.83 (1.04 – 3.20) *P* = 0.04	1.40 (1.06 – 1.85) *P* = 0.02
HIV	-	-	1.45 (0.76 – 2.76) *P* = 0.26

* Values correspond to sex- and age-adjusted *OR*s (95%*CI*s) and *P*-value

Results of multivariable logistic regression analyses showed that male sex (adjusted odds ratio, *AOR*: 1.64; 95% confidence intervals, *CI*s: 1.09 – 2.48), older age, and residing in Osh province (*OR*: 2.19, 95%*CI*: 1.05 – 4.59) were positively associated with having co-infections (see Table 3, last column).

## Discussion

To our knowledge, this is the first large-scale study that examined the seroprevalences of HBV, HCV, HIV, and *T. pallidum* among blood donors in Kyrgyzstan. A recent study on the global prevalence of chronic HBV infection classified Kyrgyzstan as a high-endemic country, with a prevalence of HBsAg of around 10% [1], but the prevalence estimate in that study was based on a single study from 1992 conducted in only one province [9]. It appears that no other estimates for HBV seroprevalence have been published since then [17]. The seroprevalence observed in our study was much lower (3.6%), which would reclassify the country from high endemicity (≥ 8%) to a country with lower intermediate endemicity (2 – 4.99%).

Regarding hepatitis C, Kyrgyzstan was classified as a country with a high prevalence in 2005 (> 3.5%) [2]. However, based on our estimate (3.1%; 95%*CI*: 3.0 – 3.3%), it would need to be reclassified into a country with moderate prevalence (1.5 – 3.5%). There has only been one study conducted on HCV prevalence in Kyrgyzstan, which was reported by Hope et al. in their review paper [17]. This study was a small-scale study conducted among pregnant women (*n* = 898) in only one province of Kyrgyzstan in 2005; the prevalence of HCV observed in this study was lower (1.6%) than the estimate from our study. Another study reported an HCV prevalence of 4% in the general Kyrgyz population [18]. Studies from the former Soviet republics showed similar HCV prevalence estimates, e.g., 2.8% (Lithuania, healthy inhabitants, *n* = 1 528) [19]; 2.9% (Russia, medical students, *n* = 173) [20]; 3% (Russia, pregnant women, *n* = 200) [21]; 3.2% (Kazakhstan, individuals aged between 10 and 64 years, *n* = 290) [22]; and 4% (Azerbaijan, healthy adults, *n* not reported) [23]. However, a few studies have reported somewhat lower prevalence estimates, e.g., 1.6% (Russia, blood donors, *n* = 3 358) [24]; 2% (Russia, blood donors, *n* = 150) [21]; 2.1% (Russia, blood donors, *n* = 4 552) [20]; and 2.2% (Lithuania, blood donors, *n* = 738) [25].

Overall, we observed a significantly decreasing trend in HBV and HCV prevalence rates over the study years. The relatively low and decreasing HBV and HCV prevalence rates could be explained by the introduction of a targeted program by the Kyrgyzstan Ministry of Health titled “Prevention and treatment of viral hepatitis in the Kyrgyz Republic for 2011–2015”, it includes, among other measures, mandatory surveillance of HBV and HCV [26], the development and implementation of goals to prevent and control HBV and HCV, and increasing access to treatment of HBV and HCV infections. There is also a recommendation for giving HBV vaccination to healthcare workers in Kyrgyzstan; however, the degree of compliance with this recommendation has not yet been assessed.

Despite this program, it is still important to undertake further activities towards the prevention and control of HBV and HCV, including surveillance of these infections, introducing HBV vaccination among high-risk groups, increasing HBV vaccination coverage among healthcare workers, and increasing awareness of HBV and in particular of HCV among healthcare workers and the general population. There are reports indicating poor access to HCV testing and treatment in the former Soviet republics, including Kyrgyzstan. The major reason for poor access to HCV treatment is its high cost. The Kyrgyz government does not fund HCV treatment and patients thus need to pay for the treatment out of pocket, which many cannot afford.

We observed a seroprevalence of HIV of about 0.78%, which is almost eight times higher than the official estimate in the general Kyrgyz population (0.1%) [10]. Moreover, we observed an increasing trend in HIV seroprevalence during the years studied. These findings are alarming and may indicate that HIV infection might shift from high-risk groups such as intravenous drug users or sex workers to the general population, and that sexual transmission may become a more frequent route of HIV transmission. This is already reflected in statistics: around 58% of HIV transmission is attributed to injecting drug use, followed by heterosexual transmission (32%, *n* = 5 113) [27]. Thus, community-based intervention programs aiming to increase HIV/AIDS-related knowledge, in particular knowledge about HIV transmission and risk factors and how to decrease sexual risk behaviors, are needed. This may include educational programs, such as school-based programs, counseling sessions, or media campaigns [28].

The prevalence of *T. pallidum* infection in the present study was 3.3%. This rate is much higher than the global (0.5%) or regional estimates of syphilis (e.g., 0.2% for the WHO European Region, 1.8% for the WHO African Region) [3]. We are not aware of any other studies that have examined the prevalence of syphilis in Kyrgyzstan. The relatively high prevalence of syphilis means there is an urgent need for the development of public health actions towards better prevention, diagnosis, and treatment of curable sexually transmitted infections (STIs).

Only a small proportion of donors (0.48%) in the total sample were co-infected with more than one of the pathogens studied. The co-infection rates in the present study ranged between less than 1% (*T. pallidum* and HIV) and 6% (*T. pallidum* and HBV). The co-infection rates observed in our study were much lower than rates reported from other regions; e.g., the rates of HIV/HBV co-infection ranged between 9 and 17% in African countries in early 2000s [29]. The HIV/HBV and HIV/HCV co-infection rates in heterosexual populations in Western Europe and USA ranged between 4 – 6% and 9 – 27% in early 2000s, respectively [30]. In our study, the risks of HBV and HCV seropositivity were almost 1.5 times higher among blood donors who were also positive for *T. pallidum*. This finding is in agreement with other studies that demonstrated that STIs increase the risk of acquiring HIV, although the effect sizes in those studies were much higher, the increase ranging between 2- and 8-fold [31].

In addition, we found that HIV seropositivity was 1.8 times higher among donors positive for HCV. This can be explained by the higher risk of transmission (either sexual, vertical, or parenteral) of these infections, which share similar modes of transmission. Although the probability of heterosexual HCV transmission is very low [32], the risk of transmission increases in the presence of HIV co-infection. For example, Nowicki et al. compared HCV RNA levels in cervicovaginal fluid of HIV/HCV co-infected and HCV monoinfected women and found that HCV RNA was present in one third of HIV/HCV co-infected women; in contrast, no HCV RNA was found among HCV monoinfected women [33]. Similar findings were found for males [34]. HIV-1 sexual transmission has been found to be higher among individuals infected with other STIs compared to individuals with no other STIs [35]. Another study showed that the odds of vertical HCV transmission were almost two times higher among women with HIV-HCV co-infection as compared to women infected with HCV alone [36]. Since these infections share similar modes of transmission, common strategies should be used to prevent their transmission.

### Limitations of the study

Although blood donors have been used to estimate the prevalence of selected infectious diseases such as HBV in the general population [1], we cannot rule out the healthy donor effect, which may result in an underestimation of true prevalence rates [6]. As mentioned above, individuals with a history of acute and chronic infections, chronic noncommunicable diseases, and risk factors of blood-transmissible infections were excluded from donating blood. In addition, our sample included both first-time (63%) and repeat donors (37%), and including repeat donors may have resulted in a lower positivity rate, as first-time donors with positive results were excluded. Furthermore, the study population had higher proportions of male and younger donors than the general population and this was not representative of the general population. However, we used poststratification weights to estimate prevalence rates in order to control for sampling bias.

In case of positive serological results, confirmatory tests (e.g., for HIV) were not performed. However, the ELISA kit used for HIV detection was a fourth generation immunoassay and the diagnostic validity of all kits was very high; for example, the sensitivity and specificity of the DS-EIA-HBsAg kit are 100 and 99%, respectively (see Table 1).

The availability of limited sociodemographic information (sex, age, province of residence, and year of blood donation) constitutes another limitation. For instance, it was not possible to assess MSM, sex worker as an occupation, and intravenous drug use as risk factors, as these constitute exclusion criteria from blood donation in Kyrgyzstan. Moreover, data on the level of education or knowledge of specific infections such as HIV/AIDS are typically not collected from blood donors in Kyrgyzstan and could not be included in the analysis.

## Conclusions

In this study, we used serological results on the presence of HBV, HCV, HIV, and *T. pallidum* among blood donors in Kyrgyzstan to estimate the respective prevalences and co-infection rates.

We observed a higher HIV prevalence than officially reported in the general Kyrgyz adult population, while the seroprevalences of HBV and HCV were lower than the available estimates. Regarding HBV endemicity, the country can thus be reclassified from high to lower intermediate endemicity. Regarding HCV, Kyrgyzstan can be reclassified from high to moderate endemicity. The high HIV prevalence with a rising trend is an alarming finding and needs to be urgently addressed by local public health authorities.

Only a small proportion of donors had co-infections. However, the risk of seropositivity (e.g., HIV, HBV, and HCV) increases with the presence of co-infections. These groups need special attention to prevent the further spread of infections and to improve therapy success by monitoring them regularly, as they are at a higher risk of developing adverse medication effects and severe liver complications.

## Additional file

Additional file 1: Multilingual abstracts in the five official working languages of the United Nations. (PDF 914 kb)

## Abbreviations

AIDS: Acquired immunodeficiency syndrome
*AOR*: Adjusted odds ratio
*CI*: Confidence interval
ELISA: Enzyme-linked immunoassay
for STI: Sexually transmitted infection
HBsAg: Hepatitis B surface antigen
HBV: Hepatitis B virus
HCV: Hepatitis C virus
HIV: Human immunodeficiency virus
MSM: Men who have sex with men
*OR*: Odds ratio
SPSS: Statistical Package for Social Sciences
WHO: World Health Organization
