# Supplementary material for: Seroprevalence of hepatitis B, hepatitis C, human immunodeficiency virus, Treponema pallidum, and co-infections among blood donors in Kyrgyzstan: a retrospective analysis (2013–2015)
Source: Infect Dis Poverty. 2017 Feb 21;6:45. doi: 10.1186/s40249-017-0255-9 (PMC5320648; doi:10.1186/s40249-017-0255-9)
Supplement: Additional file 1: — Multilingual abstracts in the five official working languages of the United Nations. (PDF 914 kb) [file 40249_2017_255_MOESM1_ESM.pdf]

Translation of the abstract into the five official working languages of the United Nations

الانتشار المصلي للالتهاب الكبدي ب، الالتهاب الكبدي الوبائي، فيروس نقص المناعة البشرية، اللولبية الشاحبة، العدوى المصاحبة بين المتبرعين بالدم في قيرغيزستان: تحليل بأثر رجعي (2013-2015)

باكيت ب. كارابايف، نورغول ج. بيشيفا، إيجانيش ب. سايتباليديفا، إيكول د. إسماعيلوفا، فرانك بسلر ماناس ك. أكماتوي

ملخص

خلفية: شهدت قرغيزستان بعد انهيار الاتحاد السوفيتي زيادة كبيرة في حالات العدوى المنقولة عن طريق الدم، ولكن هناك نقص في البيانات المدعومة بشكل كاف والدراسات الحديثة. هكذا درسنا أ) الانتشارات المصلية لمولد الضد السطحي للالتهاب الكبدي ب)، المستضد P24 لفيروس نقص المناعة البشرية النوع الأول والأجسام المضادة ضد فيروس الالتهاب الكبدي ج، فيروسات نقص المناعة البشرية (مكافحة فيروس نقص المناعة البشرية النوعين الأول والثاني، فيروس نقص المناعة البشرية النوع الأول المجموعة O، اللولبية الشاحبة بين المتبرعين بالدم في قيرغيزستان وتقييم توزيعها حسب الجنس والعمر ومحافظات الإقامة؛ ب) اتجاهات الانتشارات المصلية لها، ج) معدلات الإصابات المشتركة بين مسببات الأمراض التي شملتها الدراسة.

الطرق: تم إجراء الفحص المصلي على 37165 متبرع بالدم في مركز الدم الجمهوري في بيشكيك، قيرغيزستان، بين يناير 2013 وديسمبر 2015. طبقنا معايير الأسلوب الطبقي البعدي للسيطرة على التحيز في أخذ العينات واستعملنا تحليلات الانحدار اللوجستي لدراسة اقتران الإصابة والإصابات المصاحبة بالجنس والعمر ومحافظات الإقامة، وسنة للتبرع بالدم.

النتائج: كان 29145 (78٪) من المانحين من الذكور و8020 (22٪) من الإناث. وكان متوسط العمر 27 عاما (المدى: 18-64). معدلات انتشار مولد الضد السطحي للالتهاب الكبدي ب، واختبار الأجسام المضادة للالتهاب الكبدي ج وفيروس نقص المناعة p24 Ag واختبار الأجسام المضادة لفيروس نقص المناعة البشرية)، واختبار الأجسام المضادة للولبية الشاحبة 3.6٪ (95٪ CI: 3.4-3.8)، و3.1٪ (3.0-3.3 ٪)، 0.78٪ (0.69-0.87 ٪)، و3.3٪ (3.1-3.5 ٪) على التوالي. وكانت الذكور أكثر عرضة للإصابة بالفيروس لمولد الضد السطحي للالتهاب الكبدي ب عن الإناث (نسبة الأرجحية: 1.63، 95٪ CI: 1.40-1.90)، ولكن أقل احتمالا أن يكونوا إيجابيين مصليا لاختبار الأجسام المضادة للالتهاب الكبدي الوبائي (0.85، 0.74-0.98) وفيروس نقص المناعة البشرية (0.65؛ 0.49-0.85). وكانت معدلات الانتشار منخفضة في العاصمة مما كانت عليه في المحافظات الأخرى. كان هناك اتجاه للتناقص في الانتشارات المصلية لمولد الضد السطحي للالتهاب الكبدي ب، واختبار الأجسام المضادة للالتهاب الكبدي ج، واختبار الأجسام المضادة للولبية الشاحبة 2012-2015 (P قيمة الاتجاه، P= 0.01، P>0.0001، P>0.0001، على التوالي)، في حين ارتفع الانتشار المصلي لفيروس نقص المناعة البشرية (P = 0.049). وكان 180 من المانحين (0.48٪) إيجابيين مصليا لإصابات متعددة. لوحظ أن أعلى معدل الإصابات المشتركة بين اختبار الأجسام المضادة للولبية الشاحبة ومولد الضد السطحي للالتهاب الكبدي ب (6.0%)، يليه اختبار الأجسام المضادة للالتهاب الكبدي ج واختبار الأجسام المضادة للولبية الشاحبة (5.2%)، وفيروس نقص المناعة البشرية واختبار الأجسام المضادة للالتهاب الكبدي ج (4.9%).

الاستنتاجات: تشير البيانات إلى أن قيرغيزستان يمكن تصنيف توطن مولد الضد السطحي للالتهاب الكبدي ب ما بين المرتفع والقريب من المتوسط، في حين أن معدل انتشار فيروس نقص المناعة البشرية عال ويميل للتزايد هو اكتشاف مثير للقلق يحتاج إلى معالجة عاجلة من قبل السلطات الصحية العامة. والإصابات المصابة الملحوظة تشير إلى عوامل الاختطار الشائعة ولكن أيضا إلى تدخلات وقائية شائعة.

Translated from English version into Arabic by Mahmoud Sami, through

TRANSLATORS WITHOUT BORDERS

2013-2015 年吉尔吉斯斯坦献血者乙型肝炎、丙型肝炎病毒、人类免疫缺陷病毒、梅毒密螺旋体（*Treponema pallidum*）以及上述混合感染的血清阳性率的回顾性分析

摘要
引言：后苏联吉尔吉斯斯坦血源性感染大幅增加，但缺乏最新的、充足数据。因此，我们研究了吉尔吉斯斯坦无偿献血者中 a)乙型肝炎病毒表面抗原（HBsAg），HIV-1 p24 抗原、丙型肝炎病毒抗体（抗 HCV）、人类免疫缺陷病毒（HIV-1 / 2，HIV-1 的 O 组）抗体、梅毒螺旋体抗体的血清阳性率，同时根据性别、年龄和居住的省份评估其分布；b) 各自的血清阳性率变化趋势；c) 上述病原体的共感染率。
方法：2013 年 1 月至 2015 年 12 月，在吉尔吉斯斯坦比什凯克州血液中心对 37 165 献血者进行血清学筛查。采用分层加权抽样控制抽样偏差，采用 Logistic 回归分析评估血清阳性率和混合感染率与性别、年龄、居住省份和献血年份之间的关系。
结果: 29 145（78%）位捐献者为男性，8 020（22%）人为女性。年龄中位数为 27 岁（范围 18-64 岁）。HBsAg、抗 HCV、HIV(p24 抗原和抗 HIV)和抗梅毒螺旋体的血清抗体阳性率分别为 3.6% (95% *CI*: 3.4–3.8%)、3.1% (3.0–3.3%)、0.78% (0.69-0.87%)和 3.3% (3.1–3.5%)。男性 HBsAg 血清阳性率高于女性(*OR*: 1.63; 95% *CI*: 1.40–1.90)，但抗 HCV(0.85; 0.74–0.98) 和 HIV(0.65; 0.49–0.85)的血清抗体阳性率低于女性。首都的患病率低于其他省份。2012-2015 年，HBsAg、抗 HCV 和抗梅毒螺旋体的血清阳性率呈下降趋势（P 值趋势，$P = 0.01$，$P < 0.0001$，$P < 0.0001$），然而 HIV 抗体阳性率呈上升趋势（$P = 0.049$）。180 例捐献者（0.48%）呈多重感染。抗梅毒螺旋体和 HBsAg 的混合感染率最高，为 6.0%，其次为 HCV 和梅毒螺旋体混合感染 (5.2%)，HIV 和 HCV 混合感染率最低(4.9%)。
结论：数据表明，吉尔吉斯斯坦根据 HBsAg 的血清学阳性率可将流行区重新分为高流行区到中度流行区，而呈上升趋势的艾滋病毒感染率是一个惊人的发现，是需要公共卫生部门亟待解决的问题。出现交叉感染率表明虽存在常见的风险因素，但也有相应的常见预防干预措施。

Translated from English version into Chinese by Yin-Long Li, edited by Pin Yang, through

CHINESE CENTER FOR DISEASE CONTROL AND PREVENTION
NATIONAL INSTITUTE OF PARASITIC DISEASES

Séroprévalence de l’hépatite B, de l’hépatite C, du virus de l’immunodéficience humaine, de *Treponema pallidum* et des co-infections parmi les donneurs de sang au Kirghizstan : analyse rétrospective (2013-2015)

Bakyt B. Karabaev, Nurgul J. Beisheeva, Aiganysh B. Satybaldieva, Aikul D. Ismailova, Frank Pessler, Manas K. Akmatov

Résumé
Contexte : Le Kirghizstan postsoviétique a connu une flambée des infections transmises par le sang, au sujet desquelles il n’existe pas d’études actualisées d’une puissance suffisante. Nous avons donc étudié a) la séroprévalence de l’antigène de surface du virus de l’hépatite B (HBsAg), de l’antigène p24 du VIH-1 et des anticorps visant le virus de l’hépatite C (anti-HCV), les virus de l’immunodéficience humaine (anti-VIH-1/2, groupe O du VIH-1) et *Treponema pallidum* parmi les donneurs de sang au Kirghizstan et évalué leur distribution en fonction du sexe, de l’âge et de la

province de résidence ; b) les tendances des différentes séroprévalences et c) les taux de co-infection entre les pathogènes étudiés.
Méthodes : Un dépistage sérologique a été effectué sur 37 165 donneurs de sang au Centre du sang de la République à Bichkek entre janvier 2013 et décembre 2015. Nous avons appliqué des pondérations post-stratification pour tenir compte du biais d'échantillonnage et utilisé des analyses de régression logistique pour examiner l'association de la séropositivité et des co-infections au sexe, à l'âge, à la province de résidence et à l'année de don du sang.
Résultats : 29 145 (78 %) donneurs étaient des hommes et 8020 (22 %) des femmes. L'âge médian était de 27 ans (extrêmes : 18-64). La prévalence du VHB (HBsAg), du VHC (anticorps anti-VCH), du VIH (antigène p24 et anti-VIH) et de *T. pallidum* (anticorps) était respectivement de 3,6 % (IC à 95 % : 3,4–3,8 %), 3,1 % (3,0–3,3 %), 0,78 % (0,69–0,87 %) et 3,3 % (3,1–3,5 %). La probabilité d'être séropositifs pour l'HBsAg était plus élevée parmi les hommes que parmi les femmes (*OR* : 1,63 ; IC à 95 % : 1,40–1,90), mais plus basse pour l'anticorps anti-VHC (0,85 ; 0,74–0,98) et le VIH (0,65 ; 0,49–0,85). Les prévalences étaient plus faibles dans la capitale que dans les autres provinces. La séroprévalence de l'HBsAg et des anticorps anti-VCH et anti-*T. pallidum* a tendu à baisser de 2012 à 2015 (valeur de P pour la tendance : $P = 0,01$, $P < 0,0001$, $P < 0,0001$, respectivement), tandis que la séroprévalence du VIH a augmenté ($P = 0,049$). 180 donneurs (0,48 %) étaient porteurs de plusieurs infections. Le taux de co-infection le plus élevé concernait l'association de l'anticorps anti-*T. pallidum* et de l'HBsAg (6,0 %), suivie des anticorps anti-VHC et anti-*T. pallidum* (5,2 %) et du VIH et de l'anticorps anti-VHC (4,9 %).
Conclusions : Ces données suggèrent que l'on peut rétrograder le Kirghizstan du niveau haut au niveau bas à moyen d'endémie pour l'HBsAg, tandis que la forte prévalence du VIH et sa tendance à augmenter constituent une évolution inquiétante dont les autorités de la santé devront se préoccuper d'urgence. Les co-infections observées suggèrent des facteurs de risque communs, mais aussi la possibilité d'interventions préventives communes.

Translated from English version into French by Suzanne Assenat, through

TRANSLATORS
WITHOUT BORDERS

Исследование распространенности вирусов гепатита B и C, иммунодефицита человека и *Treponema pallidum*, а также коинфекции среди доноров крови в Кыргызстане: ретроспективный анализ (2013–2015 гг.)

Бакыт Б. Карабаев (Bakyt B. Karabaev), Нургуль Дж. Бейшеева (Nurgul J. Beisheeva), Айганыш Б. Сатыбалдиева (Aiganysh B. Satybaldieva), Айкуль Д. Исмаилова (Aikul D. Ismailova), Франк Песслер (Frank Pessler), Манас К. Акматов (Manas K. Akmatov)

Аннотация

Краткое описание. В постсоветском Кыргызстане резко выросло количество передаваемых

через кровь инфекций; однако, данных из надлежащим образом оснащенных, проведённым на современном уровне исследований недостаточно. Поэтому мы изучили a) серопревалентности поверхностного антигена вируса гепатита B (HBsAg), антигена ВИЧ-1 p24 и антител к вирусу гепатита С (анти-HCV), вирусов иммунодефицита человека (анти-ВИЧ-1/2, ВИЧ-1 группа O) и *Treponema pallidum* среди доноров крови в Кыргызстане и оценки их распределения в зависимости от пола, возраста и места проживания; b) тенденции соответствующих серопревалентностей, и c) уровни коинфекции среди исследуемых патогенов.

Методы. С января 2013 года по декабрь 2015 года была проведена серологическая проверка 37 165 доноров крови в Республиканском центре крови в Бишкеке, Кыргызстан. Мы применили постстратификацию весов для корректировки смещения выборки и метод логистической регрессии для установления зависимости серопревалентности и конинфицирования от пола, возраста, места проживания и года сдачи крови.

Результаты. Среди доноров было 29 145 (78%) мужчин и 8 020 (22%) женщин. Средний возраст составил 27 лет (диапазон: 18–64). Показатели распространения HBsAg, анти-HCV, ВИЧ (p24 Ag и анти-ВИЧ) и анти-*T. pallidum* были соответственно 3,6% (95% CI: 3,4–3,8%), 3,1% (3,0–3,3%), 0,78% (0,69–0,87%) и 3,3% (3,1–3,5%). Мужчины были с большей вероятностью серопозитивны на HBsAg, чем женщины (*OR*: 1,63; 95%*CI*: 1,40–1,90), и с меньшей вероятностью серопозитивны на анти-HCV (0,85; 0,74–0,98) и ВИЧ (0,65; 0,49–0,85). Показатели распространения были ниже в столице, чем в других районах. Наблюдалась тенденция к снижению распространённости HBsAg, анти-HCV и анти-*T. pallidum* с 2012 по 2015 год (P-значение для тенденции, $P = 0{,}01$, $P < 0{,}0001$, $P < 0{,}0001$, соответственно), в то время как распространённость ВИЧ выросла ($P = 0{,}049$). 180 доноров (0,48%) были серопозитивны на множественные инфекции. Наивысший уровень коинфекции был замечен у анти-*T. pallidum* и HBsAg (6,0%), за которыми следовали анти-HCV и анти-*T. pallidum* (5,2%), затем ВИЧ и анти-HCV (4,9%).

Заключение. Данные предполагают, что классификацию эндемичности HBsAg Кыргызстана можно изменить с высокой до пониженной промежуточной, тогда как выявленная высокая распространённость ВИЧ с тенденцией к дальнейшему росту вызывает тревогу и требует неотложных мер со стороны органов здравоохранения. Наблюдаемое коинфицирование свидетельствует о типичных факторах риска и предполагает обычные профилактические меры.

Translated from English version into Russian by Natalia Potashnik, through

TRANSLATORS
WITHOUT BORDERS

Seroprevalencia de la hepatitis B, la hepatitis C, el virus de inmunodeficiencia humana, el *Treponema pallidum*, y las coinfecciones entre donantes de sangre en Kirguistán: Un análisis retrospectivo (2013–2015)

Bakyt B. Karabaev, Nurgul J. Beisheeva, Aiganysh B. Satybaldieva, Aikul D. Ismailova, Frank Pessler, Manas K. Akmatov

Resumen

Antecedentes: En el Kirguistán postsoviético se ha observado un aumento de las infecciones de transmisión hemática, sin embargo faltan datos de estudios actualizados y con un poder estadístico adecuado. Por ello, analizamos a) la seroprevalencia del antígeno de superficie del virus de la hepatitis B (HBsAg), el antígeno p24 del VIH-1 y los anticuerpos contra el virus de la hepatitis C (anti-VHC), el virus de la inmunodeficiencia humana (anti-VIH-1/2, VIH-1 del grupo O), y el *Treponema pallidum* entre los donantes de sangre de Kirguistán, y evaluamos su distribución en función del género, la edad, y las provincias de residencia; b) las tendencias en las seroprevalencias respectivas; y c) la tasa de coinfección entre los patógenos estudiados.

Métodos: Se realizó un análisis serológico en 37.165 donantes en el centro de sangre regional (Republican Blood Centre) en Biskek, Kirguistán, entre enero de 2013 y diciembre de 2015. Se llevó a cabo una ponderación de postestratificación para controlar el sesgo de muestreo y análisis de regresión logística para examinar la relación entre la seropositividad y las coinfecciones con el género, la edad, las provincias de residencia y el año de donación de sangre.

Resultados: 29.145 (78%) donantes eran hombres y 8.020 (22%) eran mujeres. La edad media fue de 27 años (rango: 18–64). Las prevalencias del HBsAg, anti-VHC, VIH (Ag p24 y anti-VIH), y anti-*T. pallidum* fueron 3,6% (IC 95%: 3,4–3,8%), 3,1% (3,0–3,3%), 0,78% (0,69–0,87%), y 3,3% (3,1–3,5%), respectivamente. Los hombres tenían más probabilidad de ser seropositivos para HBsAg que las mujeres (*OR*: 1,63; IC 95%: 1,40–1,90), pero menos probabilidad de ser seropositivos para anti-VHC (0,85; 0,74–0,98) y VIH (0,65; 0,49–0,85). Las prevalencias fueron más bajas en la capital que en otras provincias. Se observó una tendencia decreciente en las seroprevalencias del HBsAg, anti-VHC, y anti-*T. pallidum* desde 2012 hasta 2015 (valor de *P* para las tendencias, $P = 0{,}01$, $P < 0{,}0001$, $P < 0{,}0001$, respectivamente), mientras que la seroprevalencia del VIH aumentó ($P = 0{,}049$). 180 donantes (0,48%) eran seropositivo para múltiples infecciones. La tasa de coinfección más elevada se observó entre anti-*T. pallidum* y HBsAg (6,0%), seguida de anti-VHC y anti-*T. pallidum* (5,2%), y VIH y anti-VHC (4,9%).

Conclusiones: Los datos sugieren que Kirguistán debe ser reclasificado con respecto a su endemicidad del HBsAg, pasando de elevada a una endemicidad baja-intermedia, mientras que la prevalencia del VIH con una tendencia a la alza es un dato alarmante que debe ser tratado con urgencia por los organismos de salud pública. Las coinfecciones observadas sugieren que existen factores de riesgo comunes pero también actuaciones de prevención comunes.

Translated from English version into Spanish by Barbara Gutierrez Teira, through

TRANSLATORS
WITHOUT BORDERS
